# Exploring Dynamic Metabolome of the HepG2 Cell Line: Rise and Fall

**DOI:** 10.3390/cells11223548

**Published:** 2022-11-10

**Authors:** Olga I. Kiseleva, Ilya Yu. Kurbatov, Viktoriia A. Arzumanian, Ekaterina V. Ilgisonis, Igor V. Vakhrushev, Alexey Yu. Lupatov, Elena A. Ponomarenko, Ekaterina V. Poverennaya

**Affiliations:** Institute of Biomedical Chemistry, 119121 Moscow, Russia

**Keywords:** HepG2 cell line, metabolome, GC × GC-MS, prolonged monitoring, passage number

## Abstract

Both biological and technical variations can discredit the reliability of obtained data in omics studies. In this technical note, we investigated the effect of prolonged cultivation of the HepG2 hepatoma cell line on its metabolomic profile. Using the GC × GC-MS approach, we determined the degree of metabolic variability across HepG2 cells cultured in uniform conditions for 0, 5, 10, 15, and 20 days. Post-processing of obtained data revealed substantial changes in relative abundances of 110 metabolites among HepG2 samples under investigation. Our findings have implications for interpreting metabolomic results obtained from immortal cells, especially in longitudinal studies. There are still plenty of unanswered questions regarding metabolomics variability and many potential areas for future targeted and panoramic research. However, we suggest that the metabolome of cell lines is unstable and may undergo significant transformation over time, even if the culture conditions remain the same. Considering metabolomics variability on a relatively long-term basis, careful experimentation with particular attention to control samples is required to ensure reproducibility and relevance of the research results when testing both fundamentally and practically significant hypotheses.

## 1. Introduction

Throughout the years, genomics and transcriptomics have been explored extensively for mammalian cells. However, these tools fail to measure the cell’s physiological state directly. Of all the omics layers, the metabolome is the closest to the realization of the phenotype.

Metabolome dynamically adjusts to the current physiological state of the cell, organ, or whole organism to guarantee the efficient usage of biological resources. The mechanisms of perturbations of the molecular makeup in response to internal or external changes often remain poorly understood [1]. However, the scientific community already widely accepted that metabolomic knowledge is a real “Klondike” for understanding normal and pathological molecular processes.

With the popularization of multi-omics studies (both assembled in the hands of one research group and implying the analysis of metadata deposited in public repositories), the importance of concordance and synchronicity of the obtained data is becoming more and more evident.

To gain insights into the metabolism dynamics, we investigated changes in the metabolomic profile of the HepG2 cell line. Cell lines are one of the most versatile tools for understanding living systems [2]. Due to ethical compliance, reasonable cost, and batch consistency, HepG2 has become one of the most applicable in vitro alternatives to primary human hepatocytes for a broad spectrum of biomedical projects—from cytotoxicity studies to the search for therapy for various liver cancers [3,4,5].

The general recommendation for researchers studying cell lines to answer a particular biological question is the use of early and well-annotated passages of cancer cell lines (usually up to 10–15 passages). Our work aimed to determine the significance of the HepG2 cell metabolome’s divergence after several days of continuous cultivation. The general request to evaluate the effect of prolonged cell line cultivation (under particular conditions) is reflected in several omics studies [6,7,8]. These and other projects emphasize the importance of developing some standards in the metabolomic community that consider the variability of omics data obtained during the long-term cultivation of a cell line. To estimate metabolomic stability, we compared HepG2 cell line changes on the 0th, 5th, 10th, 15th, and 20th days of cultivation. We believe that aberrations in the metabolomic profile of the HepG2 culture over time must be considered when it is used for drug metabolism testing [9], assessment of hepatocyte impairment [10], or development of potential strategies to manipulate metabolism in cancer [11]. Knowledge of such changes will allow researchers to differentiate endogenous metabolomics perturbations of the cell line from those associated with external factors or disease development.

## 2. Materials and Methods

### 2.1. Cell Culture

HepG2 cell line (human hepatoblastoma, SCC249) was purchased from Merck, Darmstadt, Germany. After thawing, the cells were cultured in DMEM/F12 supplemented with 10% fetal bovine serum and 100 units/mL penicillin/streptomycin (all from Gibco, Waltham, MA, USA) in a humidified CO_2_-incubator under standard conditions (5% CO_2_, 37 °C, 100% humidity). The medium was exchanged every three days. Upon reaching ~80% confluence, the cells were detached with trypsin-EDTA solution (PanEco, Moscow, Russia) and subcultured. Cells were grown for 5, 10, 15, and 20 days from the start of the experiment as designated. The cell cultures were observed under the Axiovert 40 CFL (Carl Zeiss, Dresden, Germany) inverted microscope and photographed with the D5000 digital camera (Nikon Inc., Tokyo, Japan). To prepare cell samples for protein extraction, the cells were detached with 0.25% trypsin-EDTA solution (PanEko, Moscow, Russia), washed three times with PBS, and counted in Goryaev’s chamber. For each time point, 5 × 10^6^ cells were harvested in two biological replicates. All operations and reagents used were maximally unified for each time-dependent cell line sample to minimize possible technical errors.

### 2.2. Flow Cytometry

For flow cytometric analysis, detached cells were resuspended in PBS and washed twice by centrifugation. FITC Annexin V Apoptosis Detection Kit I (BD Biosciences, Franklin Lakes, NJ, USA) was used to detect apoptotic and dead cells. The cells were stained with FITC Annexin V and Propidium Iodide (PI) for 15 min according to the manufacturer’s instructions.

Prior to intracellular staining, fixation and permeabilization of cells were performed using BD Cytofix/Cytoperm™ solution (BD Bioscience, Franklin Lakes, NJ, USA). One-half million permeabilized cells were stained with anti-Ki67-PE (BD Bioscience, Franklin Lakes, NJ, USA) or anti-Ki67-BV420 (Sony Biotechnology, Tokyo, Japan) monoclonal antibody. Unstained or treated with non-specific isotypic antibodies conjugated with PE samples served as negative controls. All samples were washed twice with Perm/Wash™ buffer (BD Bioscience, Franklin Lakes, NJ, USA) containing saponin. The FACSAria III flow cytometer/cell sorter (BD Bioscience, Franklin Lakes, NJ, USA) was used for the viability and Ki67 expression analyses.

### 2.3. Sample Preparation for Metabolomic Analysis

Sample preparation was performed following the protocol by O. Fiehn [12]. Briefly, intracellular metabolites were extracted using freeze–thawing cycles. Then, the low molecular weight fraction of HepG2 cells was first sequentially extracted with a mixture of isopropanol, acetonitrile, and water (3:3:2, *v*/*v*/*v*) and further with a mixture of acetonitrile and water (1:1, *v*/*v*). Next, the supernatant was evaporated to dryness in a SpeedVac evaporator (Concentrator plus, Eppendorf, Germany), then oxidized with 10 µL of freshly prepared methoxyamine hydrochloride (20 mg/mL in pyridine) at 30 °C for 90 min on a thermoshaker (ThermoMixer C, Eppendorf, Germany) at 1300 rpm. Next, the samples were derivatized by 91 µL of N-methyl-N-(trimethylsilyl)-trifluoroacetamide (MSTFA) with a mixture of fatty acid methyl esters (FAMEs) at 37 °C for 30 min at a thermoshaker at 1300 rpm. After extraction and derivatization, samples were submitted freshly for GC × GC-MS acquisition. Quality control involved reproducibility testing by monitoring the retention times and areas under the chromatographic curves of FAMEs added to both cell line and blank samples.

### 2.4. GC × GC-MS Analysis

All GC × GC-MS applications were carried out on a 7890B chromatography system (Agilent Technologies, Santa Clara, CA, USA) and a time-of-flight mass spectrometer Pegasus BT 4D (LECO Corporation, Benton Harbor, MI, USA) equipped with an L-PAL3 autosampler (CTC Analytics AG, Zwingen, Switzerland).

Each sample (1 µL) was injected through the glass liner (Restek, Bellefonte, PA, USA) under split mode (50:1). Helium (6.0 grade) was used as a carrier gas, and its constant flow of 1 mL/min was maintained throughout the run. The oven was initially heated to 60 °C, the equilibration time was 1 min, and the temperature was ramped up at the rate of 10 °C/min to the final temperature of 280 °C, with a hold time of 12 min. The first-dimension column was 30 m-long Restek Rxi-5Sil MS (Restek, Bellefonte, PA, USA, catalog #13623), and the second-dimension column was 3 m-long Restek Rxi-17Sil MS (Restek, Bellefonte, PA, USA, catalog #15123).

The transfer line of the time of MS was set at 280 °C, with a solvent delay of 350 s. The ion source temperature was 250 °C. Spectra were collected from 35–700 *m*/*z* at 70 eV electron ionization energy. The scan rate was 200 spectra per second. Data were acquired by ChromaTOF software (v. 5.51, LECO Corporation, Benton Harbor, MI, USA).

### 2.5. Data Processing

Obtained spectrum files were processed by ChromaTOF (v. 5.51, LECO Corporation, Benton Harbor, MI, USA) for deconvolution, peak picking, alignment, and primary database searching. To reduce the multi-dimensionality of the experimental data to substances that are changing between different “time points”, we used Fisher-ratio-based software ChromaTOF Tile (v. 1.01, LECO, Benton Harbor, MI, USA).

The processing principle of this software is to compare the two corresponding regions to assess the value of the difference between these 3D regions of the chromatogram (“tiles”) and to indicate low-to-high variance among tiles. We analyzed tiles (size 3 × 24 in modulation and spectra dimensions, correspondingly). Only those finds were selected for which the signal-to-noise ratio exceeded 10. The range of analyzed masses was limited to *m*/*z* = 85 and *m*/*z* = 700. Identifications were made using the components of the NIST mass spectral and retention index database (mainlib, replib) and Leco-Fiehn rtx5 library [13]. Only the hits whose forward and reverse similarity exceeded 700 were considered. After processing the initial data under the indicated conditions, in all tiles, the hits with the maximum area difference between days of cultivation (F-ratio > 5) were accepted for further analysis. F-ratio is calculated as the ratio of class-to-class variation to within-class variation. By the class, we refer to one time point containing the intensities of *m/z* features of corresponding metabolites in three technical repetitions.

Contaminants (e.g., propylene glycol, glycerol monopaltimate, and monostearate), which likely originated from laboratory plasticware or chromatography columns, were not included in the interpretation. The final list of metabolites did not include identifications, the intensity of which, in relation to the blank, on at least one of the days, was less than three. For all the remaining features, IDs and Sub-class were assigned according to HMDB (The Human Metabolome Database, https://hmdb.ca/, accessed on 5 September 2022). Next, we corrected the intensity of the selected identifications (by subtracting the corresponding intensity values in the blank samples) and normalized the results.

Statistical analyses were performed and plots were created using the R software environment (version 4.0, R Core Group, Vienna, Austria) [14]. We used multiple-way analysis of variance (ANOVA) based on multiple comparisons of means. For multiple-way ANOVA, Tukey honestly significant difference tests based on multiple comparisons of means were deployed to determine the statistically significant pairwise comparisons. Differences were considered significant if the adjusted *p*-value was less than 0.05. The pathway enrichment analysis of differentially expressed metabolites for each time point was performed using the online platform MetaboAnalyst 5.0 [15]. We matched identified metabolites with the human pathway libraries (KEGG (Kyoto Encyclopedia of Genes and Genomes [16]) and SMPDB (The Small Molecule Pathway Database [17]), and the objective metabolites and their enriched pathways were analyzed.

## 3. Results and Discussion

### 3.1. Physiological Constancy

Panoramic discovery-based metabolomic analysis generally aims to provide a comprehensive snapshot of the metabolome to illuminate certain compounds or features comprising the chemistry of the biological object under study. We characterized metabolic profiles of “time points” of the HepG2 cell line, selected on the basis of the doubling time of the culture population (ca. 2 days [18]).

To evaluate possible changes in the physiological state of cultured cells during the experiment, some critical culture characteristics were tested at days 0 and 20. After 20 days, the cell population did not change its doubling time and retained morphological features (Supplementary Figure S1a). Early (Q4) and late (Q2) apoptotic cells were almost absent at both stages of the cultivation, as can be seen from Annexin V-FITC staining (Supplementary Figure S1b). There were also no high side and low forward scatter signals common to apoptotic cells (left dot plots). The dead cells (Q1) detected by PI staining may have resulted from damaging cell dissociation for flow cytometric analysis, as the HepG2 cells tend to form aggregates. This procedure was not used to prepare samples for metabolomic analysis since obtaining a single-cell suspension was not necessary. In addition, as seen from Ki67 expression, the percentage of proliferating cells did not differ significantly at the initial and final stages (Supplementary Figure S1c). Thus, no significant changes were found in the state of the culture on days 0 and 20.

### 3.2. Metabolome Content

Methyl esters of 20 fatty acids were used as internal standards. A comparison of retention times within technical replications, and between samples from five time points, showed an RSD (relative standard deviation) of less than 0.01 s. Analysis of signal areas of each FAME on chromatograms showed that for all intraday values, coefficient of variation (CV) did not exceed 15%. CV for each FAME calculated for all technical repetitions and all time points did not exceed 3.3% (Supplementary Table S1).

**Figure 1:**
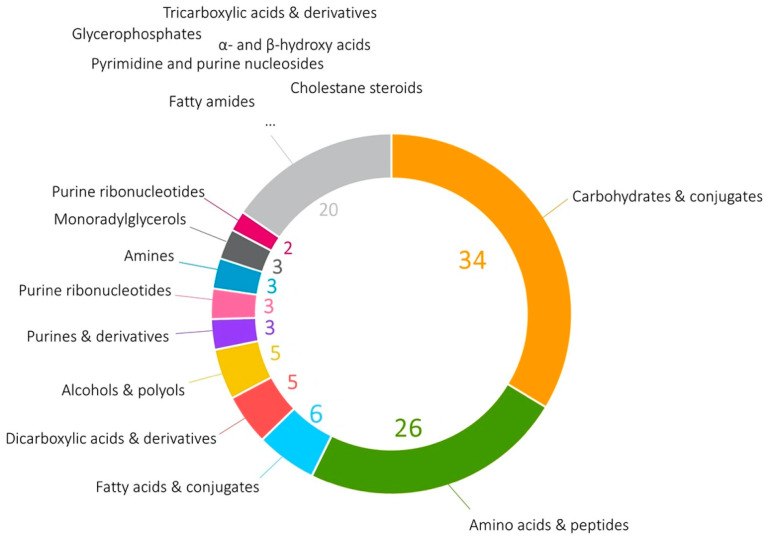
Groups of detected HepG2 metabolites according to HMDB classification. The numbers display the quantity of the discovered metabolites belonging to a certain class.

Overall, 110 unique compounds were identified in the studied samples (the full list is presented in Supplementary Table S2). Among them, the most prominent place is occupied by carbohydrates and carbohydrate conjugates (37 compounds), amino acids, peptides and their analogs (26 compounds), fatty acids (six compounds), dicarboxylic acids, and alcohols (both with five compounds, Figure 1).

The number of metabolites we detected in the HepG2 cell line is comparable or even superior to other studies using similar equipment recognized for metabolome studies [19,20].

Over-representation analysis using the hypergeometric test implemented on the MetaboAnalyst platform also highlighted that amino acids and sugars were represented more than expected by chance within the given HepG2 metabolome (see Supplementary Figure S2). However, it should be noted that the over-representation analysis of the resulting metabolome was biased by the platform of metabolome exploration used in this work, which is inherently predisposed to certain classes of compounds and corresponding molecular processes [21]. The applied derivatization procedure is effective for compounds containing amino-, hydroxy-, and carboxy-groups. Increased enrichment values for amino acids, monosaccharides, and dicarboxylic acids are in good agreement with the features of the method used.

We performed a pathway analysis on the MetaboAnalyst platform (version 5.0, Xia Research Group at McGill University, Montreal, QC, Canada) to highlight the most remarkable metabolic pathways for a more detailed consideration of the identified compounds and a better understanding of the nature of the fluctuations occurring during the cultivation period. The output of MetaboAnalyst processing was examined, and pathways with the lowest *p*-values were selected for further interpretation (see Supplementary Table S4 for a complete list of significantly enriched pathways).

### 3.3. Leading Pathways

**Figure 2:**
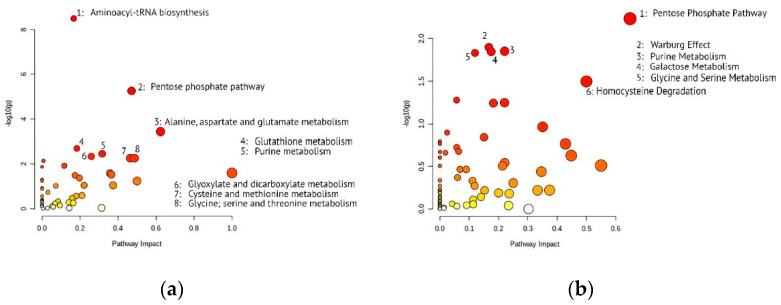
Results of pathway analysis via MetaboAnalyst against (**a**) KEGG and (**b**) SMPDB datasets in terms of *p*-value and pathway impact. Each cycle represents a pathway; its color denotes the significance level (from low—pale yellow to high—red). Most significant pathways (*p*-value < 0.01 and <0.05 for KEGG and SMPDB, correspondingly) are additionally indicated.

In the case of a study against KEGG (Figure 2a), the leaders were aminoacyl-tRNA biosynthesis (map00970), pentose phosphate pathway (map00030), glutathione (map00480), and purine (map00230) metabolism. We have also observed significant enrichment in several pathways associated with the metabolism and biosynthesis of various amino acids, e.g., alanine; aspartate and glutamate metabolism (map00250, *p*-value = 3.62 × 10^−4^). Naturally, these metabolic pathways are of great fundamental and practical interest, especially in the context of the cancer cell line [22,23,24].

Aminoacyl-tRNA biosynthesis (map00970) determines the precise match between nucleotide triplets and proper amino acids. The importance of this metabolic pathway cannot be overestimated since it provides fidelity to protein synthesis [25]. Abnormalities in protein synthesis quantity and quality of are often associated with various cancers [26], including hepatomas. The *p*-value of this pathway is 3.22 × 10^−9^, and its match status is 17/48.

The second most significantly enriched pathway, the pentose phosphate pathway (PPP, map00030), is pivotal for cancer cells. PPP is of particular interest in cancer cell line metabolome because a significant increase in glycolytic activity in the presence of abundant oxygen is one of the most striking metabolic alterations associated with oncological processes [27]. Alterations of this pathway provide a selective advantage for growth, proliferation, and survival, employing increased energy production and macromolecular biosynthesis, and counteracting oxidative stress in tumor cells. Additionally, PPP is associated with the generation of NADPH, which plays a crucial role in drug metabolism [28]. The *p*-value of this pathway is 5.51 × 10^−6^, and match status is 9/22. Interestingly, PPP and some amino acid synthesis pathways are among the most significant in the HepG2 cell line occasioned by organochlorines exposure [29].

Glutathione (gamma-glutamyl-cysteinyl-glycine) is the most abundant low-molecular-weight natural tripeptide found within almost all cells. This key metabolite in glutamine metabolism can play both healing (as a free radical scavenger) and pathogenic (through tumor progression and increased metastasis) roles. Glutathione metabolism (map00480) is associated with several processes, including cell division and proliferation. Moreover, glutathione is the most commonly elevated small molecule detected during oxidative stress [30,31]. The *p*-value is 2.05 × 10^−3^, and match status is 7/28.

All of the hallmark amino acid pathways, as well as purine metabolism (map00230, [32]), that we observed in the cell line under study, have extensive effects in cancer [33]. These metabolites may support the rapid growth of tumor cells by providing nucleotides, amino acids, and cofactors as building blocks for nucleic acid and protein synthesis, as nutrient signaling agents, neurotransmitters, epigenetic modifiers, and energy sources. In our HepG2 samples, we detected 11 of 65 metabolites involved in the purine metabolism pathway (*p*-value = 3.54 × 10^−3^). For amino acid associated pathways, the most significant were alanine; aspartate and glutamate metabolism (map00250, *p*-value = 3.62 × 10^−4^, match status = 8/28), glycine; serine and threonine metabolism (map00260, *p*-value = 5.56 × 10^−3^, match status = 7/33); and cysteine and methionine metabolism (map00270, *p*-value = 5.56 × 10^−3^, match status = 7/33).

### 3.4. KEGG vs. SMPDB Pathway Analysis Results

It is well known that the interpretation of metabolomic data is much less straightforward than that of genomic, transcriptomic, and proteomic datasets. In this regard, we duplicated the pathway analysis against another most-used database—SMPDB—and obtained slightly different results (Figure 2b, Table 1, Supplementary Table S5). This is explainable: the redundancy of identifiers and the incompleteness of databases limit the extent of the biological conclusions [34,35].

The significant pathways are partly repeated: the leaders in the search against SMPDB are also the processes of amino acid metabolism and PPP (SMP0000031)—we demonstrated this duplication in Figure 2b and in Table 1. PPP is highlighted by both SMPDB and KEGG, and it ranks first and second in significance when searching against SMPDB and KEGG, correspondingly. However, some pathways have different significance and enrichment statuses when analyzed against different databases. Several researchers have noticed this feature, which is usually associated with the unique content of pathways [35,36,37].

Thus, we observed an inconsistency in search results across different libraries for the hit-list of metabolic pathways related to aminoacyl-tRNA biosynthesis and various amino acids (alanine, aspartate, glutamate, and homocysteine). This is a notable example of the situation when the same list of detected metabolites results in discordant mapping to closely related pathways “glycine and serine metabolism” (SMP0000004, fifth most significant among identifications revealed via searching against SMPDB, *p*-value = 1.47 × 10^−2^) and “glycine; serine and threonine metabolism” (map00260, eighth most significant among identifications using KEGG, *p*-value = 5.56 × 10^−3^). The interpretation of the data obtained can be somewhat confusing in terms of reliable GC × GC-MS detection of threonine. The example of PPP can also illustrate the phenomenon of different mapping of the same metabolites. According to the results obtained from the search against KEGG, the pentose phosphate pathway contains 22 metabolites, nine of which were detected in the presented dataset. A search against the SMPDB library for the same pathway finds 8 metabolites out of a claimed 27.

Another interesting example of differences in pathway analysis is the Warburg effect [38], highlighted when searching against the background of SMPDB. This pathway ranks second among significant ones. This typical hallmark of cancer cells reflects their altered energy metabolism. The phenomenon of the Warburg effect is high rates of lactate production and glucose uptake since glucose is preferentially utilized by glycolysis rather than by oxidative phosphorylation, even in the presence of oxygen.

Due to the design of this observational experiment implicating no direct influence on the cell line, it is challenging to estimate the real significance of discordant mapping and discrepancy in the hit list of the most enriched pathways. Here, we report only the difference in pathways rating but would like to emphasize the importance of comparing the results of pathway analysis against different databases when the researcher is faced with a specific task, for example, exploring the metabolomic profile of a biological object exposed to a particular drug.

### 3.5. Dynamics

The observed enrichment in metabolic pathways with significant roles in cancer cells is anticipated concerning the object and the method of analysis. We believe that the fluctuations of the “metabolic microenvironment” during 20 days of observation are more interesting.

**Figure 3:**
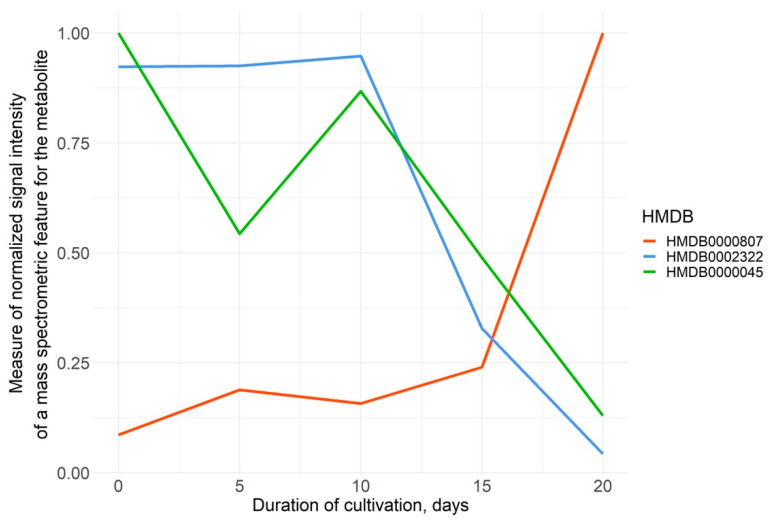
Dynamics of normalized mean intensities of *m/z* features of the corresponding metabolites (3-phosphoglyceric acid, HMDB0000807—red, cadaverine, HMDB0002322—blue, adenosine monophosphate, and HMDB0000045—green) at five time points.

Each of the detected metabolites exhibited different signal intensities between time points. For example, the intensity of *m*/*z*-features of 3-phosphoglyceric acid (HMDB0000807, which precedes serine, cysteine, and glycine and is involved in the Warburg effect [39]), increases throughout the cultivation period, with particularly active growth observed in the last five days of monitoring (Figure 3). Cadaverine (HMDB0002322), on the contrary, demonstrates a downward trend over the entire observation period. This diamine, the primary source of the putrid odor in decaying tissue, arouses interest due to its association with tumor necrosis, deriving from aggressive neoplasms. A recent study showed that cadaverine governs carcinogenesis [40]. However, the role of this metabolite is not fully understood, and there is evidence that cadaverine has a positive effect on the treatment of breast cancer through reduced metastasis [40]. Moreover, cadaverine is proposed as a potential biomarker of the effectiveness of antitumor therapy [41].

An example of chaotic changes is the behavior of adenosine monophosphate (AMP, HMDB0000045), which is used in homeostatic energy processes during high cellular energy demands [42]. Energy balance is controlled through 5′-AMP-activated protein kinase (AMPK). Evidence suggests that AMPK’s role cannot simply be defined as anti- or pro-tumor: it appears to have two faces like a double-edged sword [43], which makes studying the metabolite directly associated with the kinase even more exciting.

### 3.6. Fluctuations in Metabolites Abundancies

For a more detailed study of the dynamics of metabolites, an additional analysis of statistically significant changes in metabolites from time point to time point was carried out using multiple testing adjustments. The following adjusted *p*-values were obtained in a pairwise comparison for the metabolites detected. For most (105) metabolites, the adjusted *p*-values in at least one pair of time points were less than the confidence value of 0.05, which indicates the presence of significant intensity fluctuations between days.

**Figure 4:**
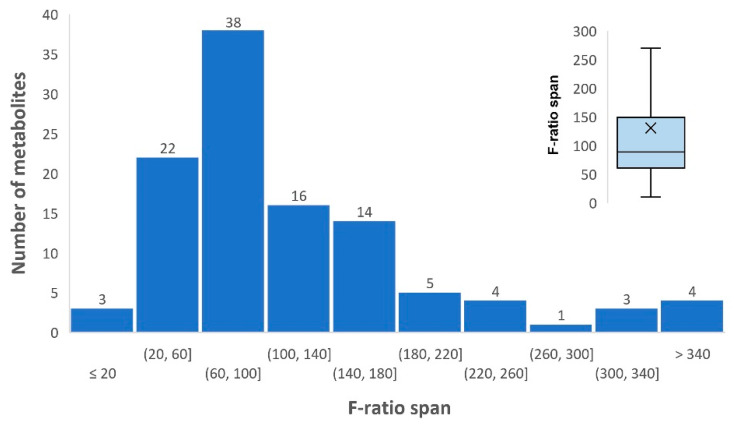
Distribution of F-ratio values for detected metabolites presented as the histogram and box-and-whiskers plot. The day of sampling serves as the X coordinate. The value along the Y axis is the normalized signal intensity *m/z* of the feature of the selected metabolite. Most of the metabolites (97%) have an F-ratio > 20, which characterizes the observed changes that we observed within 20 days as statistically significant since the critical value of F-ratio (alpha = 0.05, df1 = 5, df2 = 2) = 19.3. The cross inside the rectangle of the box-and-whiskers plot shows the mean of the F-ratios for the metabolites, and the horizontal line inside the box is the median. The horizontal dashes at the end of the “whiskers” indicate the maximum and minimum F-ratio values, excluding outliers.

The significance of the observed differences is also confirmed by the fact that for all these metabolites the value of F-ratio exceeds 10 between HepG2 cells cultured over different times (Supplementary Table S2). The distribution of averaged F-ratios for the list of identified metabolites ranges from 10 to 1630 (Figure 4). In the box-and-whiskers plot, the position of the mean and median F-ratio values relative to the total pool of values are displayed. The most (86%) metabolites are characterized by F-ratios ranging from 20 to 180.

Further study of the dynamics of the metabolite abundances led to the clusterization of metabolites into three groups according to their trends over time using the K-means approach.

The analysis of trajectories of metabolite abundances revealed a series of characteristic changes in the content of groups of low-molecular-weight substances over time. The list of changing metabolites grouped in this cluster (as well as in two subsequent clusters) is given in Supplementary Table S3.

**Figure 5:**
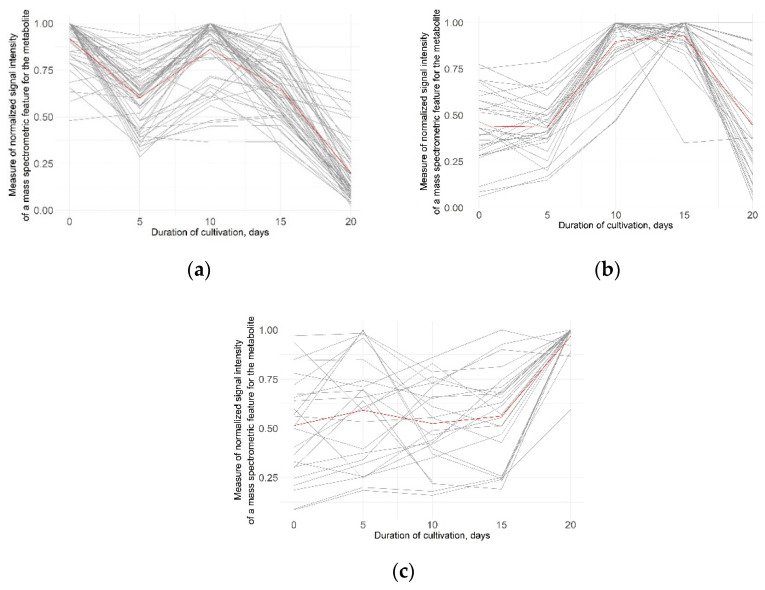
Three series of clusterization of identified metabolites (with a significant difference in abundance) using the K-means approach: (**a**) metabolites with a downward trend; (**b**) metabolites with peaking abundances at 10–15 days of cultivation; and (**c**) metabolites with zigzag fluctuations of abundances.

The first group (Figure 5a), consisting of 56 metabolites, showed a downward trend throughout cell line cultivation. This cluster is 40% carbohydrates and 20% amino acids. In addition, most of the dicarboxylic acids and alcohols found in the experiment (four of five in both cases) belong to this group.

The second group (Figure 5b) counts 32 metabolites with peaking abundance at 10–15 days of cultivation: 41% of them were presented by amino acids and analogs, 22% by carbohydrates, and both purines and amines made up 6%.

The third group (Figure 5c) of 22 metabolites demonstrates noticeable zigzag fluctuations in the first 15 days, with higher levels at the final stages. Four metabolites from this small cluster are members of the fatty acids subclass. All compounds belonging to the monoradylglycerols class are located within this cluster.

### 3.7. Affected Pathways

To test the hypothesis of co-directed regulation of metabolic pathways within a particular cluster, we again linked the metabolites inside the specific cluster to pathways according to the SMPDB and KEGG databases. We estimated their impacts via MetaboAnalyst (Table 2). The most relevant perturbed pathways in SMPDB were homocysteine degradation (SMP0000455), purine metabolism (SMP0000050), and glycolysis (SMP0000040). In KEGG those were alanine; aspartate and glutamate metabolism (map00250), aminoacyl-tRNA biosynthesis (map00970), and biosynthesis of unsaturated fatty acids (map01040). In many ways, we are witnessing an ideological repetition of the list of the most enriched metabolic pathways we found by analyzing the entire pool of reliably identified metabolites.

As expected, in certain cases, the degree of significance for the identified pathways was lower than in general in the entire dataset under study. For the pentose phosphate pathway (map00030), the *p*-value when working with the entire set of metabolites was 5.51 × 10^−6^ (KEGG). This pathway was significantly reflected in the first and second clusters. For PPP (map00030) inside the first cluster, *p*-value = 7.78 × 10^−4^ (KEGG), for the second cluster, *p*-value = 9.43 × 10^−3^ (KEGG). A similar conclusion was drawn about the decreased value of KEGG match status. For the general dataset, match status was 9/22, for the first cluster, it corresponded to 5/22, for the second, it was 3/22. It is noteworthy that this pathway, when parsing clustered data, does not appear significant, when searching against the SMPDB library.

We assume that the observed phenomenon has a biological basis because the metabolic pathway is a high-dimensional nonlinear system, with members participating in several pathways simultaneously. These metabolites form interconnected metabolic pathways that dynamically modify in frames of the whole metabolic network.

On the contrary, when moving from the general group of metabolites (match status = 5/20) to “clustered” (match status = 3/20), the glycolysis (SMP0000040) pathway slightly reduced its *p*-value from 5.72 × 10^−2^ to 8.07 × 10^−3^ (both according to SMPDB). This phenomenon may be related to the peculiarities of mathematical operations occurring inside the “black box” of MetaboAnalyst.

In addition to technical repetitions (i.e., series of measurements of the same sample showing technical noise), we repeated the metabolomic profiling of another HepG2 sample of the same starting passage to evaluate the biological variation.

To determine whether there was a significant difference between biological replicates, we examined trends in the abundance of metabolites identified in the HepG2 culture.

It was discovered that the abundances of 12% of the metabolites found in both biological repetitions (including, for example, glyceric acid, HMDB0000139, and cholesterol, HMDB0000067) change in the same direction. The behavior of 14% of core metabolites (e.g., creatine, HMDB0000064, and cadaverine, HMDB0002322) was completely non-reproducible between the first and second biological replications.

The remaining 74% of the metabolites (e.g., L-glutamine, HMDB0000641, and pyroglutamic acid, HMDB0000267) showed different abundance alteration trends, but these changes appeared in one or two time points. After the first five days of cultivation, we revealed a statistically significant change in the abundance of 15% of metabolites between the biological repetitions. We considered the change in the abundances of metabolites to be statistically significant with values of log2 (averaged abundances fold change) > 1.2 or <−1.2.

## 4. Conclusions

When studying cancer cell lines, researchers often do not specify the cultivation conditions and neglect the recommendation of using early passages [44]. Even though the lifespan of cancer cell lines is fundamentally infinite, its over-culturing presumably leads to loss of cellular identity [45]. Thus, it is essential to recognize the “unsafe moment” when the cell line does not maintain its key macro- and micro-characteristics.

Comparison of omics data is essential when searching for new molecular patterns, processing the original data, or performing a meta-analysis. However, the piecewise annotation of the object under study and the lack of understanding of the influence of the passage number on metabolomic patterns may confuse the researchers.

We attempted to get closer to the answer to the intricate question—do cells sense longitudinal monitoring with no changes in their nutrition and other treatment conditions? We investigated the stability of the metabolomic profile of the cell HepG2 line: the GC × GC-MS platform was used to assess the variability of metabolomic profiles of five samples of the HepG2 cell line, the duration of cultivation of which varied from 0 to 20 days. To avoid possible bias caused by the alignment of the cell colony along with their cycle phrases, we focused on the undisturbed mixture of several million coexisting HepG2 cells. The GC × GC-MS analysis made it possible to identify conservative metabolic patterns of the HepG2 cells and the set of metabolic features that tend to fluctuate. Such noticeable yet non-systemic shifts in the metabolome can potentially affect the interpretation of experimental results. Our conclusions comply with the findings of an impressive heterogeneity study of the HeLa cell line, demonstrating a tight connection between late passages, omics variations (including cell morphology, doubling time, karyotype, and mRNA and protein expression, as well as protein turnover rate), and fully materialized phenotypic differences [1]. The controversial moment of our conclusions may be the difference in the state of the culture medium (in other words, the availability of nutrients at different time points). However, during cultivation, we did not register physiological changes in cells. In conformity with several prolonged studies [6,7,8] performed on cell lines, we ignored desynchronization in changing the nutrient medium and fixing changes in the metabolome.

Our study showed, on the one hand, the significance of declaring the passage of the cell line when publishing results and, on the other hand, considering it in meta-analysis or testing drugs and their toxicity [9,10]. In the case of collaborations, cell passage number (or time of cultivation) and other conditions should also be kept the same [46]. Knowing the passage and conditions of cultivation will allow researchers to differentiate endogenous metabolomics perturbations of the cell line from those associated with external factors or disease development.

Further work in this area will validate the hypothesis in follow-up experiments with orthogonal approaches (e.g., LC-MS vs. GC-MS) [47] and quantify changes for normalizing data obtained at different passages. The maximum benefit for the metabolomic community will also come from research on cell line panels of a particular tumor, primary cells, organoid cultures, and pluripotent stem cells.

## Figures and Tables

**Table 1:** Ranking of the top pathway analysis results (against SMPDB and KEGG databases). The “Rank” column reflects the degree of significance of the identified pathway against both databases.

Pathway	Rank		Match Status				Raw *p*		Impact	
	SMPDB	KEGG	SMPDB		KEGG		SMPDB	KEGG	SMPDB	KEGG
			Total	Hits	Total	Hits				
Aminoacyl-tRNA biosynthesis	-	1	-	-	48	17	-	3.22 × 10^−9^	-	0.17
Pentose phosphate pathway	1	2	27	8	22	9	5.83 × 10^−3^	5.51 × 10^−6^	0.65	0.47
Alanine; aspartate and glutamate metabolism	-	3	-	-	28	8	-	3.62 × 10^−4^	-	0.62
Glutathione metabolism	28	4	19	3	28	7	3.43 × 10^−1^	2.05 × 10^−3^	0.09	0.18
Purine metabolism	3	5	63	13	65	11	1.41 × 10^−2^	3.54 × 10^−3^	0.22	0.32
Glyoxylate and dicarboxylate metabolism	-	6	-	-	32	7	-	4.64 × 10^−3^	-	0.26
Cysteine and methionine metabolism	-	7	-	-	33	7	-	5.56 × 10^−3^	-	0.46
Glycine; serine and threonine metabolism	-	8	-	-	33	7	-	5.56 × 10^−3^	-	0.49
Warburg effect	2	-	49	11	-	-	1.26 × 10^−2^	-	0.17	-
Galactose metabolism	4	15	31	8	27	5	1.42 × 10^−2^	3.28 × 10^−2^	0.18	0.17
Glycine and serine metabolism	5	-	50	11	-	-	1.47 × 10^−2^	-	0.12	-
Homocysteine degradation	6	-	7	3	-	-	3.18 × 10^−2^	-	0.50	-

**Table 2:** Overview of the top results of pathway analysis (*p*-value < 0.05) conducted against SMPDB and KEGG databases for clusterized metabolites (sorted by increasing *p*-values).

Database	Name of the Pathway	Total	Hits	Raw *p*
Cluster #1				
KEGG	Alanine; aspartate and glutamate metabolism	28	6	3.12 × 10^−4^
KEGG	Pentose phosphate pathway	22	5	7.78 × 10^−4^
KEGG	Arginine biosynthesis	14	4	1.09 × 10^−3^
KEGG	Glutathione metabolism	28	5	2.47 × 10^−3^
KEGG	Glyoxylate and dicarboxylate metabolism	32	5	4.53 × 10^−3^
SMPDB	Homocysteine degradation	7	3	4.84 × 10^−3^
KEGG	Glycine; serine and threonine metabolism	33	5	5.20 × 10^−3^
KEGG	Aminoacyl-tRNA biosynthesis	48	6	5.87 × 10^−3^
KEGG	Nitrogen metabolism	6	2	1.69 × 10^−2^
KEGG	D-Glutamine and D-glutamate metabolism	6	2	1.69 × 10^−2^
SMPDB	Methionine metabolism	39	6	1.74 × 10^−2^
SMPDB	Galactose metabolism	31	5	2.46 × 10^−2^
KEGG	Purine metabolism	65	6	2.48 × 10^−2^
KEGG	Cysteine and methionine metabolism	33	4	2.70 × 10^−2^
KEGG	Pantothenate and CoA biosynthesis	19	3	2.74 × 10^−2^
KEGG	Citrate cycle (TCA cycle)	20	3	3.14 × 10^−2^
SMPDB	Lactose Synthesis	14	3	3.81 × 10^−2^
Cluster #2				
KEGG	Aminoacyl-tRNA biosynthesis	48	10	1.19 × 10^−8^
KEGG	Phenylalanine; tyrosine and tryptophan biosynthesis	4	2	2.42 × 10^−3^
KEGG	Pentose and glucuronate interconversions	18	3	5.29 × 10^−3^
KEGG	Purine metabolism	65	5	9.29 × 10^−3^
KEGG	Pentose phosphate pathway	22	3	9.43 × 10^−3^
KEGG	Valine; leucine and isoleucine biosynthesis	8	2	1.07 × 10^−2^
SMPDB	Purine metabolism	63	6	1.17 × 10^−2^
KEGG	Phenylalanine metabolism	10	2	1.68 × 10^−2^
KEGG	Cysteine and methionine metabolism	33	3	2.87 × 10^−2^
Cluster #3				
SMPDB	Glycolysis	20	3	8.07 × 10^−3^
KEGG	Biosynthesis of unsaturated fatty acids	36	3	8.64 × 10^−3^
KEGG	Starch and sucrose metabolism	18	2	1.94 × 10^−2^
KEGG	Neomycin; kanamycin and gentamicin biosynthesis	2	1	2.44 × 10^−2^
SMPDB	Alpha linolenic acid and linoleic acid metabolism	16	2	4.53 × 10^−2^

## Data Availability

GC × GC-MS reported results are submitted to the MetaboLights repository, have permanent unique identifier MTBLS5754, and can be found at www.ebi.ac.uk/metabolights/MTBLS575 (accessed on 5 September 2022). The dataset is also available from the corresponding author on request.

## Supplementary Materials

The following supporting information can be downloaded at: https://www.mdpi.com/article/10.3390/cells11223548/s1, Table S1: Chromatographic characteristics of FAME standards: their retention times (RT, seconds), and coefficients of variation of areas under curves between technical replications and biological samples; Table S2: Metabolites detected by GC × GC-MS in HepG2 cells and averaged F-ratios for the characteristic features of a given metabolite between five time points; Table S3: Identifiers of metabolites included in the cluster 1, 2, and 3; Table S4: Pathways with the highest significance according to the MetaboAnalyst analysis against the KEGG library; Table S5: Pathways with the highest significance according to the MetaboAnalyst analysis against the SMPDB library; Figure S1: The state of HepG2 culture at the initial and final stages of its growth: (a) cell morphology and phase contrast microscopy; (b) cell viability analysis. The cells were stained with Annexin V-FITC and propidium iodide (PI) and analyzed with flow cytometry. Early (Q4) and late (Q2) apoptotic cells are almost absent at both stages of the cultivation, as can be seen from Annexin V staining. There are also no high side and low forward scatter signals common to apoptotic cells (left dot plots). The relatively high number of dead cells (Q1) detected by PI staining may result from the preparation of the cell suspension for flow cytometric analysis, as the HepG2 cells tend to form aggregates requiring dissociation. Purple color indicates the position of dead cells in forward and side light scattering coordinates; and (c) the percentage of proliferating cells. Cells were stained with one of two types of antibodies (PE or BV421 conjugated) against the marker of proliferating cells, Ki67; Figure S2: Dot plot of enriched chemical sub-classes of metabolites detected in HepG2, according to HMDB classification. Each circle denotes a sub-class, and the fill color represents the significance of enrichment of that subclass from pale yellow (low significance) to red (high significance). The enrichment *p*-value was calculated by comparing the observed frequency of metabolic hit with the frequency expected by chance. The smaller the *p*-value, the more enriched the resulting data set. The enrichment ratio is the ratio between observed and expected hits.
