# Supplementary material for: Exploring Dynamic Metabolome of the HepG2 Cell Line: Rise and Fall
Source: Cells. 2022 Nov 10;11(22):3548. doi: 10.3390/cells11223548 (PMC9688728; doi:10.3390/cells11223548)
Supplement: Supplementary file 1 [file cells-11-03548-s001.zip › cells-1929563-supplementary.pdf]

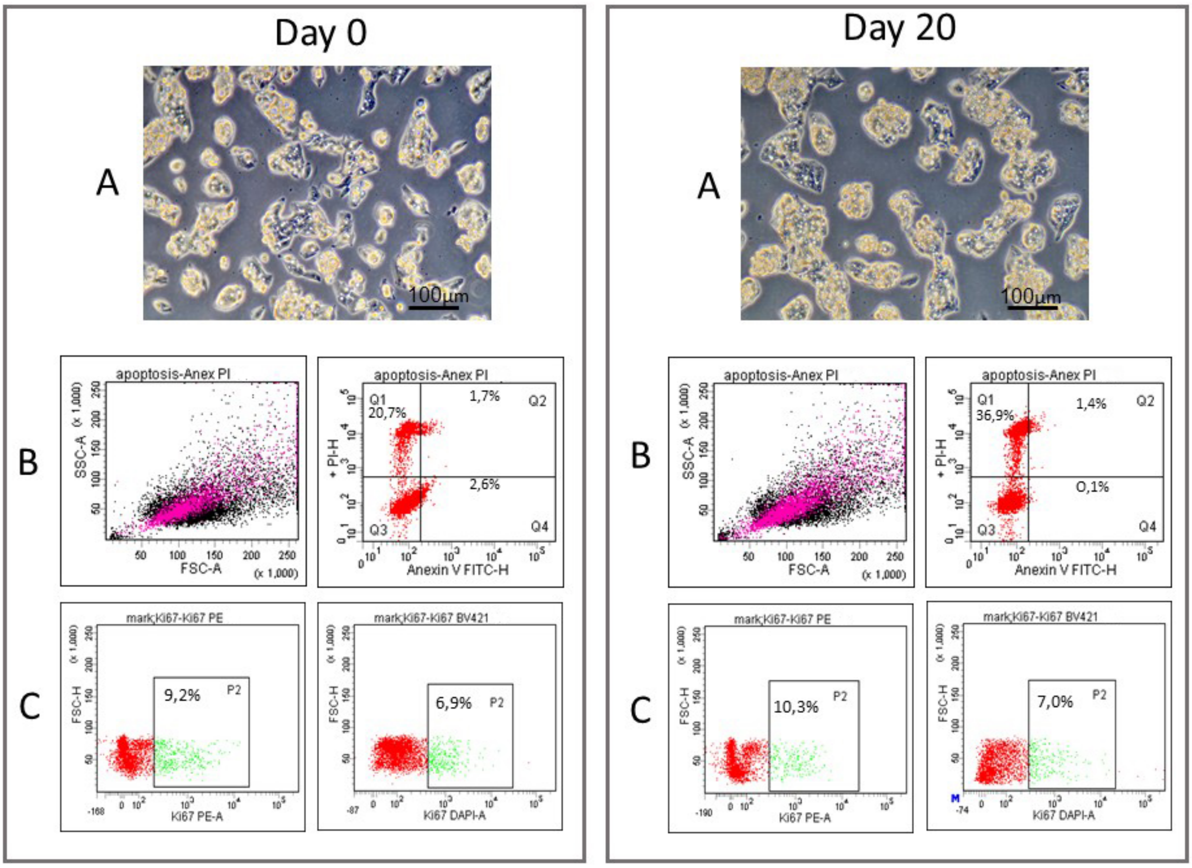

Figure S1. The state of HepG2 culture at the initial and final stages of its growth: (a) cell morphology and phase contrast microscopy; (b) cell viability analysis. The cells were stained with Annexin V-FITC and propidium iodide (PI) and analyzed with flow cytometry. Early (Q4) and late (Q2) apoptotic cells are almost absent at both stages of the cultivation, as can be seen from Annexin V staining. There are also no high side and low forward scatter signals common to apoptotic cells (left dot plots). The relatively high number of dead cells (Q1) detected by PI staining may result from the preparation of the cell suspension for flow cytometric analysis, as the HepG2 cells tend to form aggregates requiring dissociation. Purple color indicates the position of dead cells in forward and side light scattering coordinates; and (c) the percentage of proliferating cells. Cells were stained with one of two types of antibodies (PE or BV421 conjugated) against the marker of proliferating cells, Ki67;.

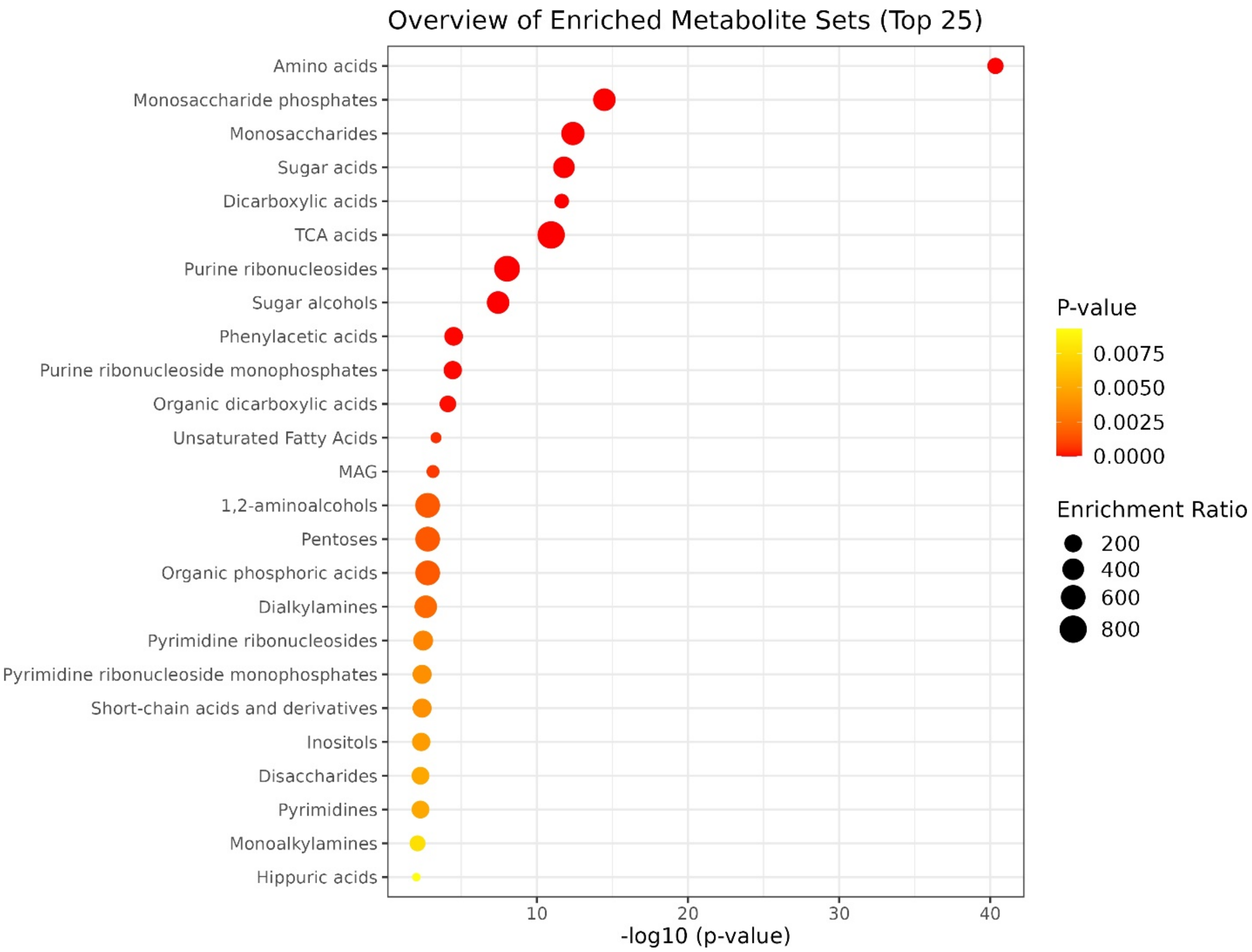

Figure S2: Dot plot of enriched chemical sub-classes of metabolites detected in HepG2, according to HMDB classification. Each circle denotes a sub-class, and the fill color represents the significance of enrichment of that subclass from pale yellow (low significance) to red (high significance). The enrichment p-value was calculated by comparing the observed frequency of metabolic hit with the frequency expected by chance. The smaller the p-value, the more enriched the resulting data set. The enrichment ratio is the ratio between observed and expected hits.

Supplementary Table S1. Chromatographic characteristics of FAME standards: their retention times (RT. seconds) and coefficients of variation of areas under curves between technical replications and biological samples

RT (s.s)		Standards	CV between technical replications, %					CV between samples. %
			0 days	5 days	10 days	15 days	20 days	
465.99	1.98	Octanoic acid. methyl ester	13.2	12.7	10.3	11.7	10.4	3.0
789.97	2.00	Dodecanoic acid. methyl ester	14.1	12.4	7.1	11.9	10.7	2.6
929.96	2.01	Tetradecanoic acid. methyl ester	13.3	12.1	10.2	11.9	9.8	1.8
985.96	2.11	(Z)-10-Pentadecenoic acid. methyl ester	12.1	11.2	10.4	11.5	9.7	1.6
993.96	2.02	Pentadecanoic acid. methyl ester	8.8	11.9	10.0	10.8	9.3	2.3
1053.95	2.04	Hexadecanoic acid. methyl ester	11.8	10.6	8.5	10.4	9.2	1.8
1113.95	2.05	Heptadecanoic acid. methyl ester	11.7	10.7	9.2	11.0	9.0	2.0
1145.95	2.28	Gamma-Linolenic acid. methyl ester	11.7	11.6	9.3	10.3	8.9	2.5
1153.95	2.15	9-Octadecenoic acid (Z)-. methyl ester	12.0	10.0	8.6	9.4	9.0	2.5
1153.95	2.22	9.12-Octadecadienoic acid. methyl ester	10.9	12.4	9.6	9.0	9.1	2.5
1157.95	2.29	9.12.15-Octadecatrienoic acid. methyl ester	12.1	7.7	8.1	12.1	9.4	3.1
1169.95	2.06	Heptadecanoic acid. methyl ester	11.8	11.5	8.7	10.1	9.5	2.2
1261.94	2.35	11.14.17-Eicosatrienoic acid. methyl ester	10.7	11.8	11.7	11.7	10.5	2.8
1273.94	2.11	Nonadecanoic acid. 18-methyl. methyl ester	12.8	11.6	9.7	10.8	10.1	1.8
1321.94	2.15	Eicosanoic acid. methyl ester	13.7	11.8	10.5	10.5	10.5	1.8
1337.94	2.62	Heneicosapentaenoic Acid methyl ester	13.1	14.9	10.1	11.1	9.8	3.3
1357.94	2.33	11.14-Eicosadienoic acid. methyl ester	14.3	12.6	10.3	11.2	13.0	3.1
1369.93	2.21	Heneicosanoic acid. methyl ester	14.0	12.2	11.1	10.9	10.6	2.6
1417.93	2.51	Docosanoic acid. methyl ester	13.5	13.0	11.0	10.9	10.5	2.5
1469.93	2.85	Hexacontanoic acid. methyl ester	13.8	12.2	10.0	12.2	9.5	2.3

Supplementary Table S2. Metabolites detected by GC×GC-MS in HepG2 cells and averaged F-ratios for the characteristic features of a given metabolite between five time points.

#	Metabolite	Derivative form	HMDB ID	F-ratio
1	p-Hydroxyphenylacetic acid	3TMS	HMDB0000020	100.5
2	5'-Methylthioadenosine	3TMS	HMDB0001173	49.6
3	Pantothenic acid	3TMS	HMDB0000210	517.1
4	myo-Inositol	6TMS	HMDB0000211	416.1
5	Inositol phosphate	5TMS	HMDB0002985	88.8
6	scyllo-Inositol	6TMS	HMDB0006088	86.5
7	2.3-Butanediol	2TMS	HMDB0003156	78.1
8	L-Lactic acid	2TMS	HMDB0000190	324.4
9	Cadaverine	4TMS	HMDB0002322	153.3
10	Ethanolamine	3TMS	HMDB0000149	25.2
11	Spermidine	1TMS	HMDB0001257	162.9
12	Creatine	3TMS	HMDB0000064	258.4
13	Cysteinylglycine	1TMS	HMDB0000078	169.8
14	L-Cystathionine	4TMS	HMDB0000099	63.3
15	L-Glutamic acid	3TMS	HMDB0000148	87.6
16	L-Threonine	1TMS	HMDB0000167	22.0
17	L-Serine	1TMS	HMDB0000187	10.1
18	Pyroglutamic acid	1TMS	HMDB0000267	92.5
19	Aminoadipic acid	3TMS	HMDB0000510	103.3
20	L-Cysteine	3TMS	HMDB0000574	20.2
21	L-Glutamine	1TMS	HMDB0000641	84.8
22	N-Acetyl-L-aspartic acid	1TMS	HMDB0000812	33.8
23	L-Aspartic acid	1TMS	HMDB0000191	52.9
24	Glycine	3TMS	HMDB0000123	44.6
25	L-Tyrosine	1TMS	HMDB0000158	108.8
26	L-Phenylalanine	2TMS	HMDB0000159	22.0
27	L-Proline	1TMS	HMDB0000162	34.6

28	L-Isoleucine	2TMS	HMDB0000172	16.7
29	L-Histidine	2TMS	HMDB0000177	105.3
30	L-Cystine	4TMS	HMDB0000192	83.6
31	L-Methionine	1TMS	HMDB0000696	27.2
32	L-Valine	2TMS	HMDB0000883	23.5
33	Aminomalonic acid	MOX, TMS	HMDB0001147	111.5
34	L-Alanine	1TMS	HMDB0000161	43.2
35	L-Norleucine	1TMS	HMDB0001645	16.6
36	2-Aminobutyric acid	2TMS	HMDB0000452	33.3
37	L-Lysine	4TMS	HMDB0000182	174.2
38	Phenylacetic acid	1TMS	HMDB0000209	21.1
39	L-Malic acid	3TMS	HMDB0000156	88.5
40	epsilon-Caprolactam	none	METPA0843	32.4
41	Melibiose	8TMS, MOX	HMDB0000048	102.4
42	Glycerol	3TMS	HMDB0000131	75.5
43	D-Maltose	8TMS, MOX	HMDB0000163	89.0
44	Alpha-Lactose	8TMS	HMDB0000186	68.8
45	Ribitol	5TMS	HMDB0000508	170.5
46	Gluconic acid	1TMS	HMDB0000625	60.2
47	Galactaric acid	6TMS	HMDB0000639	140.2
48	Rhamnose	4TMS	HMDB0000849	89.2
49	Ribonic acid	4TMS	HMDB0000867	98.0
50	Threonic acid	4TMS	HMDB0000943	107.0
51	Fructose 1.6-bisphosphate	1TMS	HMDB0001058	96.8
52	6-Phosphogluconic acid	6TMS	HMDB0001316	88.3
53	Ribose 1-phosphate	5TMS	HMDB0001489	99.4
54	Mannitol 1-phosphate	5TMS	HMDB0001530	81.3
55	Galacturonic acid	5 TMS	HMDB0002545	88.0

56	Erythrose	3TMS	HMDB0002649	87.9
57	Erythritol	4TMS	HMDB0002994	61.0
58	Deoxyribose	3TMS	HMDB0003224	147.8
59	Pectin	1TMS	HMDB0003402	78.7
60	D-Tagatose	1TMS	HMDB0003418	117.6
61	D-Glucuronic acid 1-phosphate	5TMS	HMDB0003976	97.4
62	Lactose	8TMS, MOX	HMDB0041627	84.3
63	D-Ribose	4TMS, MOX	HMDB0000283	132.1
64	D-Ribulose 5-phosphate	5TMS, MOX	HMDB0000618	141.6
65	D-Ribose 5-phosphate	5TMS, MOX	HMDB0001548	169.2
66	Glucose 1-phosphate	6 TMS	HMDB0001586	162.1
67	L-Arabitol	5TMS	HMDB0001851	142.1
68	D-Threitol	4TMS	HMDB0004136	122.4
69	Arabinofuranose	4TMS	HMDB0012325	63.6
70	D-Glucose	5TMS, MOX	HMDB0000122	169.1
71	Fructose 6-phosphate	6TMS, MOX	HMDB0000124	74.4
72	Glyceric acid	3TMS	HMDB0000139	69.6
73	D-Galactose	5TMS, MOX	HMDB0000143	270.3
74	D-Mannose	5TMS, MOX	HMDB0000169	100.8
75	N-Acetylgalactosamine	4TMS, MOX	HMDB0000212	84.7
76	3-Phosphoglyceric acid	4TMS	HMDB0000807	781.4
77	Glucose 6-phosphate	6TMS, MOX	HMDB0001401	116.1
78	Cholesterol	TMS	HMDB0000067	122.3
79	Fumaric acid	2TMS	HMDB0000134	68.4
80	Glutaric acid	3 TMS	HMDB0000661	95.2
81	Malonic acid	2 TMS	HMDB0000691	50.0
82	Oxalic acid	2 TMS	HMDB0002329	31.1
83	Succinic acid	2 TMS	HMDB0000254	57.0

84	Adipic acid	2 TMS, MOX	HMDB0000448	102.3
85	Azelaic acid	2 TMS	HMDB0000784	20.6
86	Oleic acid	1 TMS	HMDB0000207	76.7
87	Arachidonic acid	1 TMS	HMDB0001043	301.7
88	Docosahexaenoic acid	1 TMS	HMDB0002183	1630.1
89	Palmitoleic acid	1 TMS	HMDB0003229	88.3
90	Oleamide	none	HMDB0002117	61.5
91	Glycerol 3-phosphate	4 TMS	HMDB0000126	116.3
92	L-Tryptophan	2 TMS	HMDB0000929	85.4
93	MG(0:0/16:0/0:0)	2 TMS	HMDB0011533	223.3
94	MG(0:0/18:1(9Z)/0:0)	1 TMS	HMDB0011537	190.2
95	MG(18:1(9Z)/0:0/0:0)	2 TMS	HMDB0011567	184.4
96	Phosphate	3 TMS	HMDB0001429	42.9
97	Pyrophosphate	4 TMS	HMDB0000250	132.6
98	Adenosine	4 TMS	HMDB0000050	164.2
99	Inosine	4 TMS	HMDB0000195	247.7
100	1-Methyladenosine	3 TMS	HMDB0003331	31.2
101	Adenosine monophosphate	5 TMS	HMDB0000045	190.4
102	Guanosine monophosphate	6 TMS	HMDB0001397	84.4
103	Inosinic acid	5 TMS	HMDB0000175	63.6
104	Adenine	2 TMS	HMDB0000034	156.7
105	Hypoxanthine	2 TMS	HMDB0000157	323.1
106	Xanthine	3 TMS	HMDB0000292	183.3
107	Uridine	3 TMS	HMDB0000296	244.1
108	Uridine 5'-monophosphate	5 TMS	HMDB0000288	87.1
109	Uracil	2 TMS	HMDB0000300	31.3
110	Citric acid	4 TMS	HMDB0000094	191.7

TMS - trimethylsilyl group;
MOX. O-methoxyamine group

Supplementary Table S3. Identifiers of metabolites included in the clusters #1, 2 and 3.

**Cluster 1**				
HMDB0000034	HMDB0000045	HMDB0000048	HMDB0000050	HMDB0000064
HMDB0000078	HMDB0000094	HMDB0000099	HMDB0000126	HMDB0000131
HMDB0000134	HMDB0000148	HMDB0000156	HMDB0000163	HMDB0000167
HMDB0000186	HMDB0000187	HMDB0000191	HMDB0000210	HMDB0000211
HMDB0000250	HMDB0000267	HMDB0000288	HMDB0000448	HMDB0000508
HMDB0000510	HMDB0000574	HMDB0000625	HMDB0000639	HMDB0000641
HMDB0000661	HMDB0000691	HMDB0000784	HMDB0000812	HMDB0000849
HMDB0000867	HMDB0000943	HMDB0001058	HMDB0001173	HMDB0001316
HMDB0001397	HMDB0001489	HMDB0001530	HMDB0002322	HMDB0002329
HMDB0002545	HMDB0002649	HMDB0002985	HMDB0002994	HMDB0003224
HMDB0003331	HMDB0003402	HMDB0003418	HMDB0003976	HMDB0006088
HMDB0041627				
**Cluster #2**				
HMDB0000020	HMDB0000123	HMDB0000149	HMDB0000157	HMDB0000158
HMDB0000159	HMDB0000161	HMDB0000162	HMDB0000172	HMDB0000175
HMDB0000177	HMDB0000190	HMDB0000192	HMDB0000195	HMDB0000254
HMDB0000283	HMDB0000292	HMDB0000296	HMDB0000452	HMDB0000618
HMDB0000696	HMDB0000883	HMDB0000929	HMDB0001147	HMDB0001257
HMDB0001548	HMDB0001586	HMDB0001645	HMDB0001851	HMDB0003156
HMDB0004136	HMDB0012325			
**Cluster #3**				
HMDB0000067	HMDB0000122	HMDB0000124	HMDB0000139	HMDB0000143
HMDB0000169	HMDB0000182	HMDB0000207	HMDB0000209	HMDB0000212
HMDB0000300	HMDB0000807	HMDB0001043	HMDB0001401	HMDB0001429
HMDB0002117	HMDB0002183	HMDB0003229	HMDB0011533	HMDB0011537
HMDB0011567	METPA0843			

Supplementary Table S4. Pathways with the highest significance according to the MetaboAnalyst analysis against the KEGG library.

Pathway	Total	Hits	Raw p	Impact
Aminoacyl-tRNA biosynthesis	48	17	3.2197×10^{-9}	0.16667
	**List**			
	L-Asparagine; **L-Histidine**; **L-Phenylalanine**; L-Arginine; **L-Glutamine**; **L-Cysteine**; **Glycine**; **L-Aspartate**; **L-Serine**; **L-Methionine**; **L-Valine**; **L-Alanine**; **L-Lysine**; **L-Isoleucine**; L-Leucine; **L-Threonine**; **L-Tryptophan**; L-Methionyl-tRNA; 10-Formyltetrahydrofolate; **L-Tyrosine**; **L-Proline**; **L-Glutamate**; Glutaminyl-tRNA; L-Asparaginyl-tRNA(Asn); L-Seryl-tRNA(Sec); O-Phosphoseryl-tRNA(Sec); L-Histidyl-tRNA(His); L-Phenylalanyl-tRNA(Phe); L-Arginyl-tRNA(Arg); L-Cysteinyl-tRNA(Cys); Glycyl-tRNA(Gly); L-Aspartyl-tRNA(Asp); L-Seryl-tRNA(Ser); L-Valyl-tRNA(Val); L-Alanyl-tRNA; L-Lysyl-tRNA; L-Isoleucyl-tRNA(Ile); L-Leucyl-tRNA; L-Threonyl-tRNA(Thr); L-Tryptophanyl-tRNA(Trp); Tetrahydrofolate; N-Formylmethionyl-tRNA; L-Tyrosyl-tRNA(Tyr); L-Prolyl-tRNA(Pro); L-Glutamyl-tRNA(Glu); L-Glutamyl-tRNA(Gln); L-Aspartyl-tRNA(Asn); L-Selenocysteinyl-tRNA(Sec)			
Pentose phosphate pathway	**Total**	**Hits**	**Raw p**	**Impact**
	22	9	5.5058×10^{-6}	0.47103
	**List**			
	alpha-D-Glucose 6-phosphate; **Deoxyribose**; 2-Deoxy-D-ribose 1-phosphate; 2-Deoxy-D-ribose 5-phosphate; **D-Ribose 5-phosphate**; **alpha-D-Ribose 1-phosphate**; **D-Ribose**; Sedoheptulose 7-phosphate; D-Glyceraldehyde 3-phosphate; **D-Ribulose 5-phosphate**; beta-D-Fructose 6-phosphate; **beta-D-Fructose 1,6-bisphosphate**; **6-Phospho-D-gluconate**; D-Glucono-1,5-lactone 6-phosphate; beta-D-Glucose 6-phosphate; **D-Gluconic acid**; D-Glucono-1,5-lactone; **D-Glycerate**; 5-Phospho-alpha-D-ribose 1-diphosphate; D-Xylulose 5-phosphate; D-Erythrose 4-phosphate; 2-Phospho-D-glycerate			
Alanine, aspartate and glutamate metabolism	**Total**	**Hits**	**Raw p**	**Impact**
	28	8	3.6195×10^{-4}	0.6234
	**List**			
	**N-Acetyl-L-aspartate**; 2-Oxosuccinamate; **L-Aspartate**; L-Asparagine; D-Aspartate; N-(L-Arginino)succinate; N6-(1,2-Dicarboxyethyl)-AMP; **L-Alanine**; Succinate semialdehyde; **L-Glutamate**; 4-Aminobutanoate; **L-Glutamine**; Ammonia; 2-Oxoglutaramate; (S)-1-Pyrroline-5-carboxylate; N-Acetylaspartylglutamate; N-Acetylaspartylglutamylglutamate; **Citrate**; Oxaloacetate; **Fumarate**; Pyruvate; N-Carbamoyl-L-aspartate; **Succinate**; 2-Oxoglutarate; Carbamoyl phosphate; D-Glucosamine 6-phosphate; 5-Phosphoribosylamine; beta-Citryl-L-glutamate			
Glutathione metabolism	**Total**	**Hits**	**Raw p**	**Impact**
	28	7	0.0020499	0.1829
	**List**			
	R-S-Cysteinylglycine; R-S-Glutathione; Glutathione; NADP+; Glutathione disulfide; NADPH; **Glycine**; gamma-L-Glutamyl-L-cysteine; **L-Glutamate**; **L-Cysteine**; **Cys-Gly**; **5-Oxoproline**; L-Amino acid; (5-L-Glutamyl)-L-amino acid; S-Substituted L-cysteine; Acetyl-CoA; RX; L-Ornithine; Putrescine; **Spermidine**; **Cadaverine**; Tryparedoxin; Trypanothione; S-Substituted N-acetyl-L-cysteine; Spermine; Aminopropylcadaverine; Tryparedoxin disulfide; Trypanothione disulfide			
Purine metabolism	**Total**	**Hits**	**Raw p**	**Impact**
	65	11	0.0035423	0,31629
	**List**			

	Total	Hits	Raw p	Impact	List
					GDP; **Xanthine**; **D-Ribose 5-phosphate**; **L-Glutamine**; 5-Phospho-alpha-D-ribose 1-diphosphate; 5-Phosphoribosylamine; 5'-Phosphoribosylglycinamide; 2-(Formamido)-N1-(5'-phosphoribosyl)acetamidine; 1-(5'-Phosphoribosyl)-5-amino-4-imidazolecarboxamide; 1-(5'-Phosphoribosyl)-5-amino-4-(N-succinocarboxamide)-imidazole; 1-(5-Phospho-D-ribosyl)-5-amino-4-imidazolecarboxylate; 1-(5'-Phosphoribosyl)-5-formamido-4-imidazolecarboxamide; 3',5'-Cyclic AMP; ATP; ADP; dADP; **AMP**; N6-(1,2-Dicarboxyethyl)-AMP; **IMP**; **Adenosine**; dAMP; Deoxyadenosine; Deoxyinosine; Xanthosine; IDP; **GMP**; Xanthosine 5'-phosphate; **Hypoxanthine**; **Inosine**; Guanine; Deoxyguanosine; Allantoate; Guanosine 3',5'-bis(diphosphate); Guanosine 3'-diphosphate 5'-triphosphate; GTP; dGMP; dGDP; Guanosine; 3',5'-Cyclic GMP; Sulfate; Adenylyl sulfate; 5'-Phosphoribosyl-N-formylglycinamide; ITP; XTP; P1,P4-Bis(5'-adenosyl)tetraphosphate; dGTP; P1,P4-Bis(5'-xanthosyl) tetraphosphate; **alpha-D-Ribose 1-phosphate**; ADP-ribose; **Adenine**; dIDP; dITP; P1,P3-Bis(5'-adenosyl) triphosphate; dATP; 5-Hydroxy-2-oxo-4-ureido-2,5-dihydro-1H-imidazole-5-carboxylate; Urate; Aminoimidazole ribotide; Ammonia; (S)-Ureidoglycolate; Urea; 3'-Phosphoadenylyl sulfate; P1,P4-Bis(5'-guanosyl) tetraphosphate; 2'-Deoxyinosine 5'-phosphate; 5-Amino-4-imidazolecarboxyamide; (S)-Allantoin
Glyoxylate and dicarboxylate metabolism	32	7	0.0046375	0.25927	Hydroxypyruvate; cis-Aconitate; 4-Hydroxy-2-oxoglutarate; N-Formylderivatives; Glycolate; **Citrate**; **(S)-Malate**; 2-Phosphoglycolate; Acetyl-CoA; (S)-Methylmalonyl-CoA; Propanoyl-CoA; (R)-Methylmalonyl-CoA; Glyoxylate; **L-Serine**; **Glycine**; Hydrogen peroxide; **L-Glutamate**; (2S)-Ethylmalonyl-CoA; **D-Glycerate**; Acetate; 2-Hydroxy-3-oxopropanoate; Isocitrate; Pyruvate; Formate; Oxaloacetate; Acetoacetyl-CoA; Succinyl-CoA; Ammonia; Oxygen; **L-Glutamine**; (2R)-Ethylmalonyl-CoA; 2-Phospho-D-glycerate
Cysteine and methionine metabolism	33	6	0.00556	0.46344	4-Methylthio-2-oxobutanoic acid; 1,2-Dihydroxy-5-(methylthio)pent-1-en-3-one; S-Methyl-5-thio-D-ribulose 1-phosphate; S-Methyl-5-thio-D-ribose 1-phosphate; **5'-Methylthioadenosine**; S-Adenosylmethioninamine; S-Adenosyl-L-methionine; **L-Cystathionine**; **L-Serine**; L-Homocysteine; **L-Methionine**; S-Adenosyl-L-homocysteine; 2,3-Diketo-5-methylthiopentyl-1-phosphate; L-Cysteate; **L-Cystine**; **L-Cysteine**; 3-Sulfino-L-alanine; Mercaptopyruvate; (2R)-3-Sulfolactate; gamma-L-Glutamyl-L-2-aminobutyrate; **(S)-2-Aminobutanoate**; 2-Oxobutanoate; O-Phospho-L-serine; 3-Phosphonooxypyruvate; 3-(Methylthio)propanoate; Dehydroalanine; 3-Sulfopyruvate; Thiocysteine; 3-Sulfinylpyruvate; Pyruvate; 3-Mercaptolactate; Ophthalmate; 3-Phospho-D-glycerate
Glycine; serine and threonine metabolism	33	7	5.56×10^{-3}	0.48704	**L-Serine**; Choline; Betaine aldehyde; Betaine; Guanidinoacetate; 3-Phospho-D-glycerate; N,N-Dimethylglycine; **L-Cystathionine**; **Glycine**; O-Phospho-L-serine; Sarcosine; 5,10-Methylenetetrahydrofolate; **L-Threonine**; Lipoylprotein; Aminoacetone; **D-Glycerate**; [Protein]-S8-aminomethyldihydrolipoyllysine; Tetrahydrofolate; Dihydrolipoylprotein; 2-Phospho-D-glycerate; D-Serine; Hydroxypyruvate; **Creatine**; 3-Phosphonooxypyruvate; **L-Cysteine**; 2-Oxobutanoate; Glyoxylate; L-2-Amino-3-oxobutanoic acid; Pyruvate; CO2; 5-Aminolevulinate; Methylglyoxal; Ammonia
Pantothenate and CoA biosynthesis	19	5	0.0073071	0.00714	Dephospho-CoA; Pantetheine 4'-phosphate; Pantetheine; (R)-4'-Phosphopantothenoyl-L-cysteine; N-((R)-Pantothenoyl)-L-cysteine; **Pantothenate**; 3-Ureidopropionate; 5,6-Dihydrouracil; **L-Valine**; **L-Aspartate**; Apo-[acyl-carrier-protein]; CoA; D-4'-Phosphopantothenate; **L-Cysteine**; beta-Alanine; **Uracil**; 3-Methyl-2-oxobutanoic acid; Adenosine 3',5'-bisphosphate; Acyl-carrier protein

Pathway	Total	Hits	Raw p	Impact	List
Arginine biosynthesis	14	4	0.012126	0.11675	**L-Glutamate**; L-Arginine; N-Acetylornithine; N-(L-Arginino)succinate; L-Citrulline; **L-Aspartate**; Carbamoyl phosphate; L-Ornithine; Ammonia; **L-Glutamine**; 2-Oxoglutarate; N-Acetyl-L-glutamate; Urea; **Fumarate**
Valine, leucine and isoleucine biosynthesis	8	3	0.013521	0.0	**L-Threonine**; (S)-3-Methyl-2-oxopentanoic acid; L-Leucine; 3-Methyl-2-oxobutanoic acid; 2-Oxobutanoate; **L-Isoleucine**; 4-Methyl-2-oxopentanoate; **L-Valine**
Phenylalanine, tyrosine and tryptophan biosynthesis	4	2	0.025385	1.0	Phenylpyruvate; **L-Phenylalanine**; **L-Tyrosine**; 3-(4-Hydroxyphenyl)pyruvate
Phenylalanine metabolism	10	3	0.0262	0.35714	Phenylacetaldehyde; **L-Phenylalanine**; Phenethylamine; Phenylpyruvate; Benzoyl-CoA; **Phenylacetic acid**; 2-Hydroxyphenylacetate; 2-Hydroxy-3-phenylpropenoate; Hippurate; **L-Tyrosine**
Starch and sucrose metabolism	18	4	0.029991	0.36469	Cellodextrin; Cellobiose; D-Fructose; Sucrose; beta-D-Glucoside; UDP-glucose; **D-Glucose 1-phosphate**; **D-Glucose 6-phosphate**; D-Glucose; Amylose; alpha,alpha-Trehalose; Maltodextrin; Starch; **Maltose**; Dextrin; Isomaltose; **D-Fructose 6-phosphate**; alpha-D-Glucose 1,6-bisphosphate
Galactose metabolism	27	5	0.03275	0.17315	Stachyose; D-Tagatose 6-phosphate; D-Gal alpha 1->6D-Gal alpha 1->6D-Glucose; Sucrose; Raffinose; Melibiose; D-Galactose; 3-beta-D-Galactosyl-sn-glycerol; Epimelibiose; Melibiitol; alpha-D-Galactosyl-(1->3)-1D-myo-inositol; alpha-D-Glucose; **Lactose**; **D-Glucose 1-phosphate**; UDP-alpha-D-galactose; UDP-glucose; alpha-D-Galactose 1-phosphate; **alpha-D-Galactose**; alpha-D-Glucose 6-phosphate; D-Tagatose 1,6-bisphosphate; D-Glucose; D-Fructose; Galactitol; **Glycerol**; D-Mannose; D-Sorbitol; **myo-Inositol**
Citrate cycle (TCA cycle)	20	4	0.042744	0.19704	Enzyme N6-(dihydrolipoyl)lysine; 2-Oxoglutarate; Thiamin diphosphate; Enzyme N6-(lipoyl)lysine; 3-Carboxy-1-hydroxypropyl-ThPP; Succinyl-CoA; **Succinate**; Oxalosuccinate; Isocitrate; Oxaloacetate; Acetyl-CoA; **(S)-Malate**; cis-Aconitate; **Citrate**; Pyruvate; 2-(alpha-Hydroxyethyl)thiamine diphosphate; [Dihydrolipoyllysine-residue succinyltransferase] S-succinyldihydrolipoyllysine; **Fumarate**; [Dihydrolipoyllysine-residue acetyltransferase] S-acetyldihydrolipoyllysine; Phosphoenolpyruvate

Supplementary Table S5. Pathways with the highest significance according to the MetaboAnalyst analysis against the SMPDB library.

Pathway	Total	Hits	Raw p	Impact
Pentose Phosphate Pathway	27	8	0.0058294	0.64907
	**List**			
	Beta-D-Glucose 6-phosphate; NADP; Gluconolactone; NADPH; Water; **6-Phosphogluconic acid**; **D-Ribulose 5-phosphate**; Carbon dioxide; **D-Ribose 5-phosphate**; **D-Ribose**; Adenosine triphosphate; ADP; **Ribose 1-phosphate**; Phosphoribosyl pyrophosphate; Adenosine 2'-phosphate; Xylulose 5-phosphate; D-Glyceraldehyde 3-phosphate; D-Sedoheptulose 7-phosphate; D-Erythrose 4-phosphate; **Fructose 6-phosphate**; **Fructose 1,6-bisphosphate**; Dihydroxyacetone phosphate; Hydrogen phosphate; Acetaldehyde; Deoxyribose 5-phosphate; Deoxyribose 1-phosphate; **Glucose 6-phosphate**			
Warburg Effect	**Total**	**Hits**	**Raw p**	**Impact**
	48	11	0.012627	0.29145
	**List**			
	Alpha-D-Glucose; Adenosine triphosphate; **Glucose 6-phosphate**; ADP; **Fructose 6-phosphate**; **Fructose 1,6-bisphosphate**; Dihydroxyacetone phosphate; D-Glyceraldehyde 3-phosphate; NADH; Hydrogen phosphate; Glyceric acid 1,3-biphosphate; **3-Phosphoglyceric acid**; 2-Phospho-D-glyceric acid; Phosphoenolpyruvic acid; Water; Pyruvic acid; Hydrogen carbonate; Oxalacetic acid; Coenzyme A; Acetyl-CoA; Carbon dioxide; **Citric acid**; Ammonium lactate; Hydrogen Ion; (1R,2R)-Isocitric acid; Oxoglutaric acid; Succinyl-CoA; Guanosine diphosphate; **Succinic acid**; Guanosine triphosphate; Ubisemiquinone; FAD; **Fumaric acid**; QH(2); FADH; **L-Malic acid**; D-Glutamic acid; Ammonia; D-Glutamine; D-Erythrose 4-phosphate; D-Sedoheptulose 7-phosphate; **D-Ribose 5-phosphate**; **D-Ribulose 5-phosphate**; 6-Phosphonoglucono-D-lactone; **6-Phosphogluconic acid**; Hydrogen; Beta-D-Glucose 6-phosphate; NADP; NADPH			
Purine Metabolism	**Total**	**Hits**	**Raw p**	**Impact**
	13	63	0.01406	0.22087
	**List**			
	Diguanosine tetraphosphate; Water; Guanosine triphosphate; **Guanosine monophosphate**; Adenosine triphosphate; Guanosine diphosphate; ADP; Hydrogen phosphate; dGDP; dGTP; 2'-Deoxyguanosine 5'-monophosphate; Deoxyguanosine; **Pyrophosphate**; Guanine; Phosphoribosyl pyrophosphate; Guanosine; **Ribose 1-phosphate**; Deoxyribose 1-phosphate; **Xanthine**; Ammonia; Adenosine diphosphate ribose; Adenosine 2'-phosphate; **D-Ribose 5-phosphate**; 5-Phosphoribosylamine; D-Glutamic acid; D-Glutamine; **Glycine**; Glycineamideribotide; 10-Formyltetrahydrofolate; Tetrahydrofolic acid; 5'-Phosphoribosyl-N-formylglycinamide; Phosphoribosylformylglycineamidine; 5-amino-1-(5-phospho-D-ribosyl)imidazole-4-carboxylate; 5-Aminoimidazole ribonucleotide; Carbon dioxide; **L-Aspartic acid**; SAICAR; **Fumaric acid**; 5-Aminoimidazole-4-carboxamide; Phosphoribosyl formamidocarboxamide; **Inosinic acid**; Xanthylic acid; NADH; NADP; NADPH; Xanthosine; **Inosine**; **Hypoxanthine**; Oxygen; Hydrogen peroxide; Uric acid; Deoxyinosine; 6-Succinoaminopurine; **Adenosine**; **Adenine**; Deoxyadenosine; Cyclic AMP; Deoxyadenosine monophosphate; dADP; Deoxyadenosine triphosphate; Guanosine 2',3'-cyclic phosphate; IDP; Inosine triphosphate			
Galactose Metabolism	**Total**	**Hits**	**Raw p**	**Impact**
	8	31	0.014242	0.17516
	**List**			
	L-Galactose; Sorbitol; Melibiitol; **D-Mannose**; Epimelibiose; **myo-Inositol**; Galactinol; **Glycerol**; Galactosylglycerol; Stachyose; Raffinose; Sucrose; **Alpha-Lactose**; **Melibiose**; Water; D-Fructose; Galactan; NADH; L-Iditol; Adenosine triphosphate; Galactose 1-phosphate; ADP; Uridine diphosphategalactose; Alpha-D-Glucose; Uridine 5'-diphosphate; **Glucose 1-phosphate**; Uridine diphosphate glucose; **Pyrophosphate**; Uridine triphosphate; **Glucose 6-phosphate**; Hydrogen phosphate			
Glycine and Serine Metabolism	**Total**	**Hits**	**Raw p**	**Impact**
	11	50	0.01472	0.11953
	**List**			

	Aminoacetone; Oxygen; Water; Ammonia; Hydrogen peroxide; Pyruvaldehyde; NADH; Pyruvic acid; L-2-Amino-3-oxobutanoic acid; Carbon dioxide; 2-amino-3-ketobutyrate coenzyme A ligase, mitochondrial; **Glycine**; Dimethylglycine dehydrogenase, mitochondrial; Formaldehyde; 8-[(Aminomethyl)sulfanyl]-6-sulfanyloctanoic acid; Aminomethyltransferase, mitochondrial; Dihydrolipoate; 5,10-Methylene-THF; Serine hydroxymethyltransferase, mitochondrial; **L-Serine**; Dihydrolipoyl dehydrogenase, mitochondrial; **Serine--pyruvate aminotransferase**; D-Alanine; Serine--pyruvate aminotransferase; **Glycine amidinotransferase, mitochondrial**; Guanidoacetic acid; **Guanidinoacetate N-methyltransferase**; S-Adenosylmethionine; 5-aminolevulinate synthase, nonspecific, mitochondrial; 5-Aminolevulinic acid; Betaine--homocysteine S-methyltransferase 1; Homocysteine; Racemethionine; **Glycine N-methyltransferase**; **Glycerate kinase**; Adenosine triphosphate; **3-Phosphoglyceric acid**; ADP; Phosphohydroxypyruvic acid; Phosphoserine aminotransferase; DL-O-Phosphoserine; Oxoglutaric acid; **Phosphoserine phosphatase**; Hydrogen phosphate; Adenosine 2'-phosphate; **Pyrophosphate**; L-Seryl-tRNA(Ser); Glycyl-tRNA(Gly); **L-Cystathionine**; 2-Ketobutyric acid			
Homocysteine Degradation	**Total**	**Hits**	**Raw p**	**Impact**
	3	7	0.031817	0.5
	**List**			
	Homocysteine; **L-Serine**; Water; **L-Cystathionine**; **L-Cysteine**; 2-Ketobutyric acid; Ammonia			
